# Survey of Methylmercury Exposures and Risk Factors Among Indigenous Communities in Guyana, South America

**DOI:** 10.5696/2156-9614-10.26.200604

**Published:** 2020-05-04

**Authors:** L. Cynthia Watson, Jorge L. Hurtado-Gonzales, Christopher J. Chin, Juliana Persaud

**Affiliations:** 1 Watershed Hydrology and Ecology Research Division, Environment and Climate Change Canada, Burlington, Ontario, Canada; 2 Sustainable Environments and Social Solutions, Ontario, Canada; 3 World Wildlife Fund, Guianas, Guyana Office, Georgetown, Guyana

**Keywords:** indigenous people, South America, toxicity, artisanal and small-scale gold mining, 2020

## Abstract

**Background.:**

Gold mining activities in forested areas across Guyana have been a common practice for more than a century. The intensification of artisanal and small-scale gold mining (ASGM) in recent decades caused by global market demand is contributing to the mobilization of mercury into aquatic systems. Indigenous populations who consume high levels of locally sourced fish are greater at risk for methylmercury poisoning from ingestion of contaminated fish.

**Objectives.:**

The aim of the present study was to investigate the levels of mercury contamination and identify the risk factors associated with hair mercury levels in four indigenous communities in Guyana.

**Methods.:**

Concentrations of total mercury were measured in hair samples from 99 participants from four indigenous communities in the south Rupununi region in Guyana. The findings of this study were compared with those of previous studies to assess the prevalence of mercury contamination in indigenous communities across Guyana.

**Results.:**

Hair mercury levels were found to be above the World Health Organization (WHO) reference value for residents who live close to ASGM activities and who consume high quantities of locally sourced fish. Our results are not only consistent with those obtained in previous studies, but also evidence that mercury poisoning has become a generalized problem for indigenous communities in Guyana.

**Conclusions.:**

Fish is the main source of protein for many riverine communities and consumption of mercury-contaminated fish poses a serious health hazard for these vulnerable populations. The situation is especially dire for community members of Parabara with 100% of participants showing elevated (>15 μg*g^−1^) hair mercury levels. It is therefore crucial that Parabara residents be evaluated by relevant health agencies for clinical symptoms related to mercury toxicity.

**Participant Consent.:**

Obtained

**Ethics Approval.:**

The study protocol was approved by the Institutional Review Board of the Ministry of Public Health, Guyana.

**Competing Interests.:**

The authors declare no competing financial interests.

## Introduction

The gold mining industry in Guyana has experienced unprecedented growth in the last two decades to the extent that it now dominates the export industry and has become one of the country's most important economic sectors. In 2017, the export of raw gold alone accounted for 13% of the country's GDP and 56.8% of its exports.^1^,^2^ This growth, fueled by global demand, is contributing to the release of record amounts of mercury into the environment and elevated rates of deforestation. Deforestation in Guyana has progressed to the point that the price of gold has become one of its best predictors.^3^

Gold mining in Guyana is dominated by the artisanal and small-scale gold mining (ASGM) sub-sector and accounts for 70% of the country's total gold production (according to Bulkan and Palmer there is no distinction between small- and medium-scale mining in Guyana, so in this article, the term “small-scale” includes small- and medium-scale operations).^4^,^5^ Artisanal and small-scale gold mining operations rely heavily on metallic mercury for gold recovery and this is one of the key pathways through which mercury enters freshwater systems in Guyana.^6^ For 2008 alone, mercury use for gold amalgamation approximated 15 000 kg, and increased to 35,820 kg in 2013.^7^ Considering only the official amounts of declared annual gold production, it can be estimated that approximately 1 kg of mercury was used to amalgamate 1 kg of gold in 2008 and 2.39 kg for each kg of gold in 2013.^7^ The high dependence on mercury by the ASGM sector was captured in an analysis of geochemical data obtained along the Essequibo and Mazaruni Rivers, which suggests that high amounts of mercury have already been mobilized into aquatic systems.^6^ Furthermore, high levels of mercury have also been detected in geochemical samples from areas where gold mining activities are not legally allowed or where mining is not known to have occurred.^8^

Forest clearing for mining operations represents another major pathway through which mercury is released into aquatic systems. Deforestation contributes to soil erosion which in turn enhances mercury mobilization.^9^ In addition to deforestation, gold mining activities cause major impairment to rivers and streams. River dredging is especially destructive as it requires drilling into riverbeds and banks and moving large quantities of soil. It is estimated that ASGM activities have caused damage to 5 840 km of rivers and streams and potentially impacted an additional 28 771 km of downstream habitat with turbidity and mercury.^10^

The Guyanese government is working towards the implementation of responsible mining in the gold industry, yet gold extraction as it is practiced has adverse impacts on the ecosystem, health, and wellbeing of Guyanese indigenous people.^11^–^13^ First, alluvial gold-rich areas are located in zones that are in dispute or occupied by indigenous communities.^4^,^5^,^11^–^16^ Second, gold mining has not been a viable solution to the economic hardships experienced by indigenous communities who face barriers in access to adequate health care, education, and employment opportunities.^12^,^13^ Some communities experience greater health disparities (e.g., alcoholism, domestic abuse, rape, child prostitution, illicit drug use) despite the increased income to the local economy.^15^–^17^ Third, because of their heavy reliance on local resources, indigenous people are exposed to higher levels of methylmercury through the consumption of contaminated fish.^18^–^20^

Methylmercury is one of the most toxic forms of mercury and elevated levels can cause irreversible damage, reducing intellectual and motor capacity, especially in the developing fetus and breastfed children.^21^,^22^ Methylmercury poisoning presents a range of other symptoms, but the main effects are concentrated in the nervous, digestive, renal, respiratory, and cardiovascular systems.^21^,^22^

In Guyana, mercury contamination in indigenous populations close to ASGM activities has been reported since the late 1990s. Studies by Singh *et al.* and Couture *et al.* have reported high levels of mercury in both people and fish, with carnivorous fishes showing higher levels than non-carnivorous fishes.^18^,^20^ Furthermore, Couture *et al.* found higher levels of mercury in fishes from all three trophic levels (herbivores, omnivores, and carnivores) sampled from mining areas compared to non-mining areas.^18^ Although some efforts have been made to educate miners and limit the use of mercury in ASGM activities, mercury exposure remains a serious and continuous risk for indigenous populations located close to mining activities.^23^

Concerned about the impacts of gold mining, the South Central People's Development Association–a community organization representing indigenous communities in south Rupununi–requested an investigation into mercury exposure in community members through discussions with officers of the World Wildlife Fund, Guyana. Following this request, our team developed a study to measure mercury levels and identify risk factors (i.e., age, gender, location of residence, duration of residence, education level, and frequency of fish consumption) associated with methylmercury levels in hair samples in four communities in south Rupununi. Results were then compared with those previously obtained by non-governmental organizations from several communities within Guyana.^18^–^20^,^24^

**Abbreviations**
ASGM: Artisanal and small-scale gold mining
GLM: Generalized linear model

## Methods

**Figure 1:**
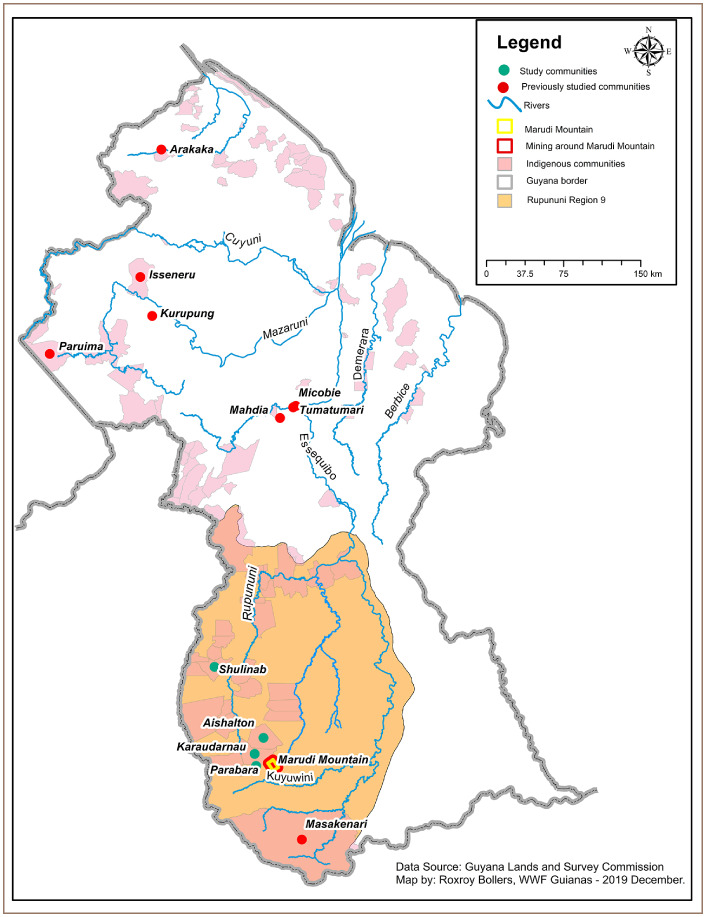
Map of Guyana showing the study communities and previously studied communities used for comparison purposes

There are 17 indigenous communities in the south Rupununi region at varying distances from mining areas and with varying numbers of community members involved in mining activities. The Rupununi region is located in southwest Guyana and the Rupununi River flows south to north through this region *(Figure 1).* The communities of Aishalton, Karaudarnau, and Parabara were selected to assess the levels of mercury contamination in community members and the community of Shulinab was selected to serve as a control. The study communities are relatively close to one another and to the Marudi mining site, between 30 and 40 km, and residents make use of the Rupununi and Kuyuwini Rivers *(Figure 1).* The Kuyuwini River experiences high levels of turbidity likely from upstream mining activities, whereas the Rupununi River does not appear to be impacted by mining activities.^8^ The control community of Shulinab is located a considerable distance from the other three communities and any active mining (~ 140 km) in south Rupununi.

A major portion of Parabara residents' protein supply comes from fish sourced from the Kuyuwini River *(Figure 1).* Karaudarnau residents also consume a diet high in local fish, but most of it is captured from the Rupununi River. The communities of Aishalton and Shulinab consume a small quantity of locally sourced fish, and cattle and poultry are the major sources of protein for these two communities. Information about food sources was gathered during reconnaissance visits to the communities.

### Sample and data collection

The study was conducted from March to May 2017. Prior to the implementation of the study, a team consisting of South Central People's Development Association representatives and a member of the research team visited and held meetings with community leaders and members. The study objectives and relevance of community participation were outlined and discussed during the meetings. Following this visit, each village council provided a letter endorsing the study and their willingness to participate. At the national level, permission was obtained from the Ministry of Indigenous Peoples' Affairs and the study protocol was approved by the Institutional Review Board of the Ministry of Public Health, Guyana.

Ninety-nine individuals volunteered to participate in the study (58 females and 41 males). Participants' age ranged from 15 to 78 years (mean ± SD: 36.80 ± 15.44). The mean age for female participants was 34.31 ± 13.85 years (mean ± SD) and 40.31 ± 16.80 years for male participants. Most of the participants had been living in their communities for an average of 25.29 ± 16.05 years. The average household size was 6 persons (45% males, 41% females), and 12% with children less than 5 years old. In terms of education, 64% of the participants had a primary education or lower. Parabara and Karaudarnau had a higher percentage of participants with primary or fewer years of education. Only 36% of the participants attended high school.

Prior to data collection, each study participant was provided with a verbal description of the project by a member of the research team. Volunteers were then given the option to decline or participate in the study. Once an individual agreed to participate, they were required to provide written consent by signing a consent form. Each participant was then interviewed and hair samples were collected by a member of the research team. Translation was done by bilingual field assistants for participants who were not literate in English. Information collected included sociodemographic information (age, gender, education level, occupation, duration of residence, etc.) and rate of fish consumption (e.g., daily, weekly, monthly intake).

### Mercury in hair

From each participant, 4 cm long hair strands were cut close to the scalp in the occipital region and collected. The sample was divided/cut into two segments: 0–2 cm and 2–4 cm, placed in separate labeled Ziploc bags, and stored in the dark under ambient temperature until analysis for mercury. The first 2 cm closest to the scalp (0–2 cm) was used to determine total mercury content. Human hair grows at a rate of 0.6 to 3.36 cm per month and using the first 2 cm of hair closest to the scalp can identify if participants were recently exposed to elevated levels of mercury.^25^ Using mercury concentration found in newly formed hair reflects the relative amount of mercury already present in the blood stream.^26^ Mercury that accumulates in hair is not only easy to measure, but remains stable, and is easy to transport and store.^27^

Twenty-five samples of the first 2 cm of hair were randomly selected and subsampled (Shulinab: 6 people, Aishalton: 6 people, Parabara: 6 people, and Karaudarnau: 7 people) to determine methylmercury hair content. A regression analysis was performed considering the total mercury hair content and methylmercury hair content using the 25 subsamples. The results indicated a strong linear relationship between total mercury and methylmercury indicating that 98.31% (±1.27) of total mercury in hair was in the form of methylmercury. This result supports the validity of measuring total mercury as a proxy for methylmercury in hair.

Mercury hair content analyses were performed by a certified laboratory (ALS Inc., Burlington, Ontario, Canada Branch). The lab, ALS, used sample digestion via United States Pharmacopeia 233 and United States Environmental Protection Agency (USEPA) Method 7470A to eliminate elemental impurities.^28^,^29^ Mercury analysis was then performed via cold vapor atomic absorption technique using Method 7470A.^28^ Three different readings were taken per sample and then averaged to estimate the total mercury concentration. Quality control included a strict blank control and calibration curve before sample batches (20 samples per batch) were analyzed. The detection limit for total mercury in hair was 0.005 μg*g^−1^.

### Mercury concentration in human hair recommended limits

A level of 1 to 2 μg*g^−1^ for mercury found in hair is considered to be normal; however, people who consume fish on a daily basis are expected to have levels exceeding10 μg*g^−1^.^30^ The Joint Food and Agricultural Organization (FAO)/World Health Organization (WHO) Expert Committee on Food Additives has set a provisional tolerable weekly intake of 1.6 μg methylmercury per kg of body weight, which is equivalent to a total hair mercury concentration of 2.5 μg*g^−1^.^31^,^32^ This reference limit is considered to protect developing fetuses from the harmful effects of mercury exposure. In the present study, 2.5 μg*g^−1^ of mercury in hair was used as a reference level for our study populations as proposed by the WHO.^22^,^31^,^32^

### Statistical analyses

The distribution of total mercury levels found in hair samples at each study location along with the spatial distribution associated with the frequency of fish consumption are both represented in the form of box plots*.* Each box plot represents the first quartile (lower hinge), median, and third quartile (upper hinge). The whiskers are the upper and lower values. The circular markers represented by nodes outside of the box plots are individual values (outliers) that are outside of the range of adjacent values. Wherever indicated, the significance of sample differences was tested using a Mann-Whitney two-sample mean comparison test to a 95% confidence level.

A generalized linear model (GLM) using maximum likelihood optimization of a linear model was fit to mercury hair levels. This model aimed to determine whether gender, location of residence, duration of residence, education level, and frequency of fish consumption represented immediate risk factors associated with total mercury measurements found in hair. All these variables may offer a more complete explanation of the recent exposure to total mercury values observed in hair samples. The confidence level of the GLM was set to 95%. All statistical analyses were performed using JMP^®^ version 13.

## Results

**Figure 2:**
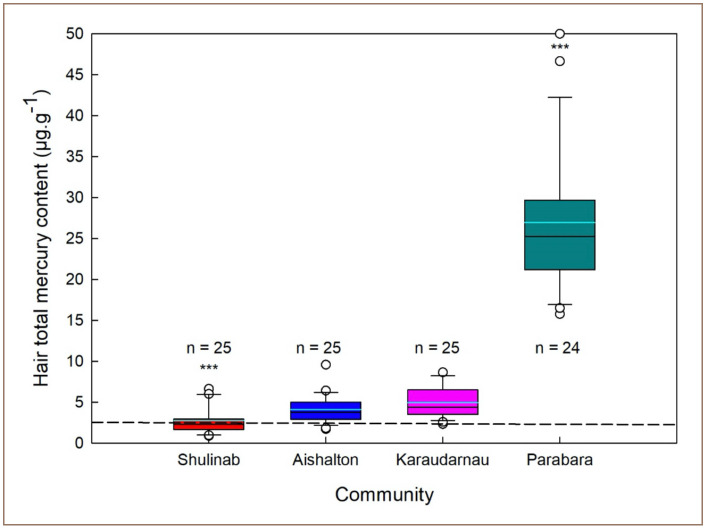
Mercury levels in hair samples across study communities The dashed line indicates the reference limit of 2.5 μg*g^−1^

Mean concentration and frequency distributions of total mercury in hair samples of residents from the communities of Shulinab, Aishalton, Karaudarnau, and Parabara are presented in Figure 2. The average total mercury value was 9.84 ± 3.82 μg*g^−1^ (n = 99), with the lowest and highest values equal to 0.87 μg*g^−1^ (Shulinab) and 50 μg*g^−1^ (Parabara), respectively. Mean total mercury levels decreased with increasing distance from the main mining site in south Rupununi: Parabara (27.62 ± 10.22 μg*g^−1^); Karaudarnau (4.89 ± 1.98), Aishalton (4.03 ± 1.50) and Shulinab (2.74 ± 1.63 μg*g^−1^).

### Risk factors associated with methylmercury measurements in hair

The GLM effect test indicated that age was a non-significant risk factor (p> 0.05). Thus, we re-ran the GLM, where age was treated as a covariate. With age as a covariate, the GLM test showed that the difference between log-likelihoods for the full and reduced models is 92.83, with a p-value < 0.001, which indicates that the model as a whole is significant. The location of residence and frequency of fish consumption were shown to be the two most important risk factors for total mercury levels found in hair of south Rupununi residents *(Table 1).* Gender, education, and duration of residence did not show any significant effect on the levels of mercury in hair *(Table 1).*

**Table 1:** Relationship Between Socio-demographic Factors and Risk of Elevated Mercury Levels in Hair in Study Communities

**Explanatory variables**	**DF**	***x^2^***	**P**
Location of residence	3	25.18	<0.001*
Frequency of fish consumption	2	19.01	<0.001*
Gender	1	0.22	0.64
Duration of residence	2	0.35	0.56
Education level	3	2.47	0.48

Abbreviation: DF, degrees of freedom.

* Significant p value.

**Generalized linear model with normal Distribution, Identity as link function**

### Location of residence

The GLM showed that residence location is one of the most important variables for mercury found in hair of south Rupununi residents (p < 0.0001). In particular, total mercury levels in hair of people from the community of Parabara were found to be significantly higher than people living in the communities of Karaudarnau, Aishalton, and the control community of Shulinab (all p< 0.001) *(Figure 2).* The control community of Shulinab differed significantly with regard to total mercury levels in hair from the communities of Aishalton (Mann-Whitney U = 148, n=25, n=25; p = 0.01), Karaudarnau (Mann-Whitney U = 97, n=25, n=25; p < 0.001) and Parabara (Mann-Whitney U = 0, n=25, n=24; p < 0.001). No statistical difference was found between the communities of Aishalton and Karaudarnau (both Mann-Whitney tests p > 0.1).

### Frequency of fish consumption

**Figure 3:**
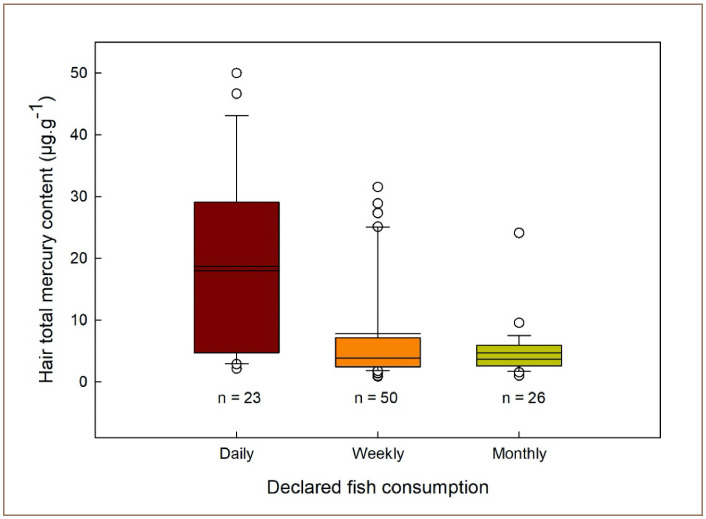
Hair total mercury content according to frequency of declared fish consumption

Frequency of fish consumption was a significant predictor of mercury hair levels in the present study (p < 0.0001) *(Table 1 and Figure 3).* Intake frequencies were calculated as the number of times a participant reported eating fish: daily, weekly, or monthly. Fifty-six percent (56%) of all participants reported eating fish daily and this percentage increased to 100% for the community of Parabara, indicating that Parabara residents receive the majority of their protein from locally sourced fish.

### Gender

**Figure 4:**
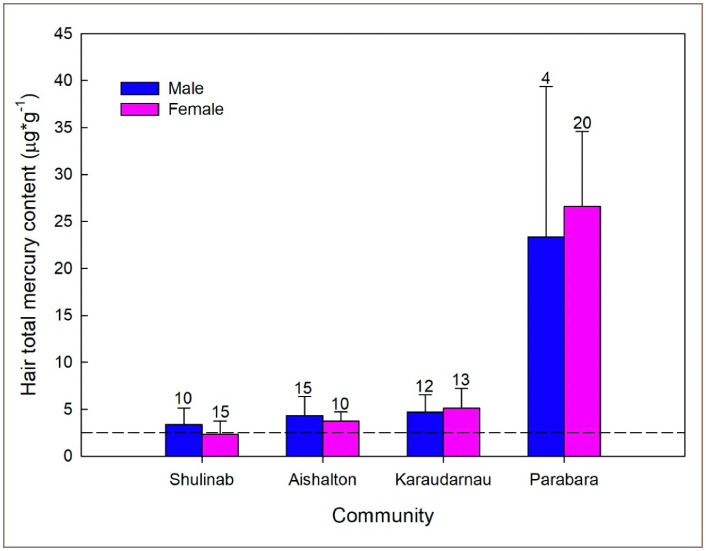
Mean total mercury concentration in hair from south Rupununi residents by gender. *The numbers above SD bars represent the number of participants for each gender group for each community. ~The dashed line indicates the reference limit of 2.5 μg*g^−1^.

The GLM suggests that gender is not an important risk factor with respect to mercury exposure (p > 0.05) in this study. However, it is important to consider that in the community with the highest levels of hair mercury contamination, women in Parabara had the highest levels of mercury (average of 28.65 μg*g^−1^), while men in that community had an average mercury level of 26.58 μg*g^−1^ *(Figure 4).*

### Duration of residence

Although years of residency varied widely across communities, duration of residence was not an important predictor in terms of recent exposure to total mercury (p > 0.05). The analysis failed to capture any difference in the duration of residence in people from communities like Parabara, where 100% of the participants migrated from another community, primarily Masakenari (a non-mining community). Participants' residency time varied between >2 months to 30 years, with an average of 13.01 years, which indicates that their migration was relatively recent. In contrast, almost 69% of participants from the communities of Shulinab, Aishalton, and Karaudarnau were born in those communities.

## Discussion

**Figure 5:**
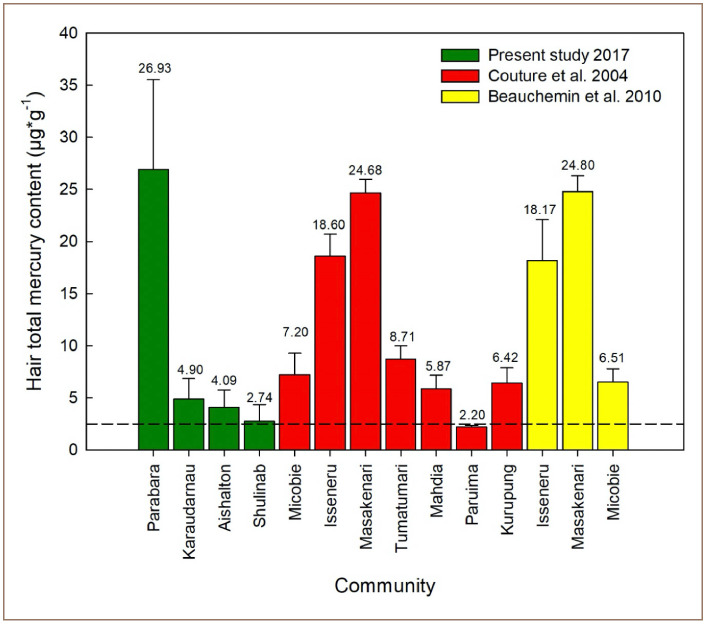
Reported average hair mercury level content in fish-eating communities in south Rupununi, Lower Potaro River, Mazaruni River, Upper Essequibo River, Kurupung River, Barima River, and Kamarang River *Means are expressed on the top of standard deviation (SD) bars. The dashed line indicates the reference limit of 2.5 μg*g^−1^.

The findings of the present study indicate that indigenous people in the south Rupununi region living close to ASGM activities and who consume high levels of locally sourced fish are likely to show high concentrations of total mercury. In particular, people from the community of Parabara expressed a record high average level of 26.93 μg*g^−1^ total mercury. This result is comparable only to the levels of total mercury found in the Guyanese indigenous people of Masakenari reported by Couture *et al.* and Beauchemin *et al.* and are well above other previously studied communities *(Figure 5).*^18^,^19^ The people of Parabara also show higher levels of total mercury contamination than any other indigenous communities previously studied in neighboring countries (i.e., Venezuela, Suriname, French Guiana; see also Legg *et al*.).^33^ In contrast to Parabara, the communities of Karaudarnau, Aishalton, and Shulinab, which are further from ASGM activities, showed low levels of mercury contamination *(Figure 2 and Figure 4).* The low levels of mercury found in people from these communities may be explained by the fact that these communities have access to alternative sources of protein (i.e. poultry and beef).

Numerous studies across South America have shown that riverine communities are highly dependent on fish as their main source of protein, exceeding 400 g/person/day and as high as 600 g/person/day.^33–38^ Communities that do not have access to other sources of protein face a dilemma, having to choose between eating contaminated fish or eating a diet practically devoid of protein, and this decision is usually based on the lack of other resources and purchasing power. Couture *et al.* measured total mercury in hair in several indigenous communities across Guyana *(Figure 5)* and found the highest levels in the riverine communities of Isseneru (18.60 μg*g^−1^; Mazaruni River) and Masakenari (24.68 μg*g^−1^; Upper Essequibo River).^18^ These findings were associated with the frequent consumption of contaminated carnivorous fish species. Likewise, Beauchemin *et al.* assessed total mercury concentrations in hair from pregnant and lactating women living in the communities of Micobie, Isseneru, and Masakenari.^19^ The results demonstrated that indigenous women of reproductive age (15–45 years old) from the communities of Isseneru and Micobie harbored total mercury values as high as 45.7 and 70.8 μg*g^−1^, respectively. The community of Masakenari showed an average level of 24.80 ± 1.5 μg*g^−1^ *(Figure 5).* These results consistently show that communities that rely heavily on locally sourced fishes are likely to express high levels of total mercury in hair samples.

The reference value for adults should be revised because it might not reflect the physiological responses of indigenous people in the Amazon and there is no consensus on exactly what constitutes a proper reference (e.g., Dorea *et al.*, Ashe, Langeland *et al.*).^39–43^ This rationale is based on the differences between feeding profiles of indigenous communities in the Amazon basin and communities from other geographical regions.^40^ Recent studies in the Tapajos region in Brazil indicated that the translocation of mercury from soils to the upper parts of cultivated and wild plants is negligible, and therefore the consumption of fruits and plants should not represent any health hazards.^44^ Due to the soluble dietary fiber content and prebiotic nutrients provided by local fruits, it is possible that demethylation of methylmercury occurs, reducing their levels of toxicity.^39^

Additionally, it is important to evaluate the balance between the benefits of nutrients such as omega-3 fatty acids and the risks of methylmercury exposure from fish. Freshwater fish species provide important nutrients, particularly polyunsaturated fatty acids.^45^ These nutrients contribute to brain and visual system development in infants and help to reduce the risks of certain forms of heart disease in adults. Thus, a realistic framework for providing dietary advice for women of childbearing age on maximizing dietary intake of polyunsaturated fatty acids while minimizing methylmercury exposures should be developed focusing on behavior change approaches.

At this stage and taking into consideration that Guyana has ratified the Minamata Convention on Mercury–a global treaty aimed at protecting human health and the environment from the adverse effects of mercury emissions due to anthropogenic industrial activities–the community of Parabara should be cognizant of the results and initiate discussions regarding alternative sources of protein or reduced consumption of carnivorous fishes. We acknowledge that this will be challenging because of their local tradition and lack of alternatives, but it is important to recognize that high levels of mercury may compromise the health of residents. It is critical to investigate if there is an association between high levels of bioaccumulated mercury expressed in hair samples and clinical symptoms due to the frequent consumption of contaminated fish.^46^ It is important to consider whether it is realistic to assume that mercury concentrations ≥ 2.5 μg*g^−1^ jeopardizes the health of local residents. Further rigorous studies to develop reference limits in Guyana's hinterland are needed to orient viable risk management strategies to reduce exposure while maintaining a healthy fish diet.

Mercury biomonitoring in indigenous populations to identify those with high levels of mercury exposure should be implemented. Continued biomonitoring for methylmercury exposure is recommended, including follow-up studies to monitor trends, which are especially important in riverine communities close to ASGM activities.^47^ More importantly, in order to safeguard future generations, there is a need to implement short and long-term studies to assess mercury exposure in children less than 6 years of age, particularly for indigenous infants and children, given that no data exist for Guyana.

Permit granting and regulatory agencies (such as the Guyana Geology and Mines Commission and Environmental Protection Agency) should employ better land-use planning and consult with local communities when granting permits for gold mining activities to ensure that such activities do not negatively impact critical freshwater resources and other areas used by indigenous populations. This is with the aim of starting to develop measures for safer ASGM practices including mercury phase-out in the south Rupununi region and other mining areas across Guyana. Additionally, it is important for the relevant agencies to start taking into account the potential effects of deforestation and other extractive activities that contribute to the removal of forest cover which facilitates the release of mercury into water bodies and the atmosphere.

As a final note, it is important to mention that the overall results were presented to community leaders and members in their respective communities. A technical report was also provided to community leaders and relevant government agencies.

## Conclusions

The present study showed that indigenous populations in Guyana who live close to artisanal and small-scale gold mining activities and who depend on local fish to supply most of their protein needs are likely to harbor high levels of mercury in their bodies. The situation is especially dire for community members in Parabara. One hundred percent of adult participants from Parabara showed hair mercury levels >15 μg*g^−1^. It is therefore crucial that Parabara residents be evaluated for clinical symptoms related to mercury toxicity by the relevant health agencies in Guyana. In addition, it is necessary to develop a plan so that community members can be continuously informed of the adverse effects of methylmercury and how to balance fish intake without compromising their health.
